# MicroRNAs Are Mediators of Androgen Action in Prostate and Muscle

**DOI:** 10.1371/journal.pone.0013637

**Published:** 2010-10-27

**Authors:** Ramesh Narayanan, Jinmai Jiang, Yuriy Gusev, Amanda Jones, Jeffrey D. Kearbey, Duane D. Miller, Thomas D. Schmittgen, James T. Dalton

**Affiliations:** 1 Preclinical Research and Development, GTx, Inc., Memphis, Tennessee, United States of America; 2 College of Pharmacy, The Ohio State University, Columbus, Ohio, United States of America; 3 Lombardi Cancer Center, Georgetown University, Washington, D. C., United States of America; New Mexico State University, United States of America

## Abstract

Androgen receptor (AR) function is critical for the development of male reproductive organs, muscle, bone and other tissues. Functionally impaired AR results in androgen insensitivity syndrome (AIS). The interaction between AR and microRNA (miR) signaling pathways was examined to understand the role of miRs in AR function. Reduction of androgen levels in Sprague-Dawley rats by castration inhibited the expression of a large set of miRs in prostate and muscle, which was reversed by treatment of castrated rats with 3 mg/day dihydrotestosterone (DHT) or selective androgen receptor modulators. Knockout of the miR processing enzyme, DICER, in LNCaP prostate cancer cells or tissue specifically in mice inhibited AR function leading to AIS. Since the only function of miRs is to bind to 3′ UTR and inhibit translation of target genes, androgens might induce miRs to inhibit repressors of AR function. In concordance, knock-down of DICER in LNCaP cells and in tissues in mice induced the expression of corepressors, NCoR and SMRT. These studies demonstrate a feedback loop between miRs, corepressors and AR and the imperative role of miRs in AR function in non-cancerous androgen-responsive tissues.

## Introduction

Nuclear hormone receptors represent the largest family of ligand-activated transcription factors and play pivotal roles in diverse biological functions[1]. For example, androgen (AR) and estrogen (ER) receptors are essential for reproduction, bone, and muscle development, while glucocorticoid receptor regulates inflammation and glucose homeostasis[2]–[4] These roles make the drugs that target nuclear hormone receptors as one of the largest classes of drugs[5].

The long-held belief that nuclear hormone receptors mediate the pharmacologic actions of hormones solely through direct DNA binding has unraveled over the last decade revealing an even more complex signaling cascade.

AR is known to regulate gene expression through direct DNA binding as well as through protein-protein interaction[6], [7]. AR interacts with coactivators and corepressors, which are large classes of proteins that augment or suppress receptor function, respectively[7]–[9]. These proteins are critical for AR function and knockout of these proteins produce a wide range of phenotypes in animal models[10].

microRNAs (miRs) are short 22 nucleotide non-translatable RNAs that bind to the 3′ untranslated region (UTR) of target genes and repress or degrade mRNAs. miRs are synthesized as primary miRs (pri-miRs) by RNA Pol II, which are converted to mature miRs by the RNAse enzymes, Drosha and DICER[11], [12].

In the last few years, there is increasing inquisitiveness regarding the importance of miRs in normal development and pathological transformation of tissues[13]–[16]. Moreover, due to their presence in serum and owing to their stability, miRs are being evaluated as biomarkers for the early detection of several diseases including cancer and obesity[17]–[19]. Despite these developments, ligand- or drug-dependent miR regulation and their significance in nuclear hormone receptor function has not been demonstrated clearly.

Recent studies in mammalian tissues and drosophila indicated the involvement of miRs in nuclear hormone receptor function[20], [21]. Yamagata *et al* showed that estrogen-ER-α complexes interacted with Drosha and down-regulated the expression of a subset of miRs leading to altered ER function[20]. Similar studies, utilizing *in vitro* characterization in prostate cancer cells, identified androgen responsive elements (AREs) in the promoter of miR-21 and miR-125b[22], [23].

In this study, we investigated the importance of miRs in AR function. Androgens up-regulated the expression of a large set of miRs in prostate and levator ani muscle in rats. Tissue-specific knockout of Dicer in mice completely impaired AR function leading to an androgen-insensitivity syndrome. This work clearly demonstrates that miRs are mediators of AR function and the existence of a possible feedback loop between miRs, AR and corepressors.

## Materials and Methods

The animal studies were conducted under the guidance and approved protocols of the Animal Care and Use Committee (ACUC) of the University of Tennessee. The pharmacodynamic study was performed as published earlier under the approved protocol (approval ID 1673) of the ACUC of University of Tennessee. The Dicer knockout study was performed under the approved protocol (approval ID 1763) of the ACUC of University of Tennessee.

### Animal pharmacodynamic experiment

The pharmacodynamic study was performed as published earlier under the approved protocol (approval ID 1673) of the Animal Care and Use Committee of University of Tennessee [6]. Briefly, five male Sprague Dawley rats per group (250 g) from Harlan (Indianapolis, IN) were treated subcutaneously for 14 days with 3 mg/day of DHT, SARM-1, SARM-2, SARM-3 or vehicle. Dosing solutions were prepared daily by dissolving the drugs in dimethyl sulfoxide (DMSO) and diluting in polyethylene glycol 300 (PEG 300). At the end of 14 days, the animals were sacrificed, weights of prostate and levator ani measured and the tissues collected for RNA isolation.

#### RNA Analysis and reverse transcriptase Polymerase Chain Reaction

LNCaP cells were plated at 10,000 cells per well of a 96 well plate in RPMI supplemented with 1% csFBS or in full serum. The cells were maintained for 3 days and were treated with vehicle, or AR ligands. RNA was isolated using cells-to-ct kit (Applied Biosystems) and the expression of various genes were measured using TaqMan primer probe mix (Applied Biosystems) on an ABI 7900 realtime PCR machine. The expression of individual genes was normalized to GAPDH levels. For siRNA experiments, LNCaP cells were plated at 10,000 cells/well in Accell siRNA transfection medium and transfected with Accell DICER- or cyclophilin -siRNA (Dharmacon Inc., Lafayatte, CO).

### Co-Immunoprecipitation

Cell extracts were prepared in homogenization buffer (0.05 M potassium phosphate, 10 mM sodium molybdate, 50 mM sodium fluoride, 2 mM EDTA, 2 mM EGTA, and 0.05% monothioglycerol [pH 7.4] containing 0.4 M NaCl and protease inhibitors [1 mg each of aprotinin, leupeptin, antipain, benzamidine HCl, and pepstatin/ml], 0.2 mM phenylmethylsulfonyl fluoride, and 1 mM sodium vanadate) by three freeze-thaw cycles. Immunoprecipitation was carried out as follows. Briefly, 100 µl of a 1∶1 slurry of protein A-Sepharose beads in 1× TE (0.01 M Tris and 0.001 M EDTA) was incubated for overnight at 4°C with 5 µg of DICER-1 antibody (DICER A-2 mouse monoclonal antibody from SantaCruz biotechnology). The beads were washed with 1× TE, and incubated overnight at 4°C with 100 µg of protein extract in 400 µl of the lysis buffer without salt. The beads were then washed for 5 min once each with high-salt buffer (0.1% sodium dodecyl sulfate [SDS], 1% Triton X-100, 2 mM EDTA, 20 mM Tris HCl [pH 8.1], 0.5 M NaCl), low-salt buffer (same as high-salt wash buffer but with 0.15 M NaCl), and 1× TE (10 mM Tris HCl, 1 mM EDTA; pH 8.0). The immunoprecipitated proteins were extracted with 2× Laemmli buffer, separated on an SDS-polyacrylamide gel electrophoresis (SDS-PAGE) gel, and the AR band detected by Western blotting.

#### Chromatin Immunoprecipitation Assay (ChIP)

ChIP assays were performed as described previously[24]. Briefly, proteins were cross-linked by incubation with 1% formaldehyde (final concentration) at 37°C for 10 min and immunoprecipitated with 5 µg of NCoR or SMRT antibody (Santacruz Biotechnology) and protein A sepharose, rotating overnight at 4°C. DNA-protein complexes were obtained by extracting the beads with 50 µl of freshly prepared extraction buffer (1% SDS, 0.1 M NaHCO_3_) three times. Cross-linking of the DNA protein complexes was reversed by incubating at 65°C for 6 h. The DNA was extracted with a QIAquick PCR purification kit (QIAGEN, Valencia, CA) in 25 µl final volume of TE and the recruitment was detected by realtime PCR using primers and probe described earlier[6].

### Serum RNA isolation

Total RNA was purified from rat serum using Qiagen miRNeasy Mini Kit with modifications (Qiagen, Valencia, CA). Briefly, 200 µl of serum was homogenized in 800 µl of Qiazol Lysis reagent, and mixed with 160 µl of chloroform. Eleven fmol each of two synthetic C. elegens miRNAs was spiked into the aqueous phase before precipitation with 1.5 volumes of 100% ethanol. Total RNA was eluted in 40 µl of RNase-free water.

### Reverse transcription and qPCR for tissues

Total RNA (500 ng) was isolated using Qiagen RNA extraction columns and primed for 312 mouse miRNAs and 4 reference genes using the TaqMan microRNA Assay protocol (Applied Biosystems). Reverse transcription was performed as follows: 40 cycles of 16°C for 2 min/42°C for 1 min/50°C for 1 sec followed by 85°C for 5 min. cDNA was diluted 50-fold and 1 µL was used in a 5 µL qPCR to assay the 312 miRNAs and 4 reference genes in 384 well plates using standard conditions (single replicate). Data are presented as 2^−ΔC^_T_ using 18S rRNA as the reference gene.

### Reverse transcription and qPCR for serum

The total RNA that was eluted from the Qiagen miRNeasy column (3.75 µl) was primed for 312 mouse miRNAs, 2 C. elegans miRNAs and 4 additional reference genes in a 15 µl reaction using the TaqMan MicroRNA Assays (Applied Biosystems). Reverse transcription was performed by 40 cycles of 16°C 2 mins/42°C 1 min/50°C 1 sec/followed by 85°C for 5 mins. The cDNA (2.5 µl) pre-amplified in a 25 µl reaction following the Applied Biosystems Megaplex pre-amplification protocol for microRNA. Pre-amplification was performed at 95°C/10 min, 55°C/2 min and 72°C/2 min and then 12 cycles of 95°C/15 sec and 60°C/4 minutes. The pre-amplified cDNA was diluted 1∶4 in 0.1X TE buffer (pH 8.0), a aliquot of which was further diluted 1∶50 and 1 µL was used in a 5 µL qPCR using standard conditions to profile 312 mouse miRNAs and the 6 reference genes (single replicate). Data are presented as 2^−ΔC^_T_ using the mean of the 2 C. elegans spike in oligos as a reference.

### Statistics and Ingenuity pathway analysis

For downstream statistical analysis, normalized expression data were imported in TIGR MeV[25]. Permutations based Students T-tests[26] and ANOVA[27] were performed to determine differentially expressed miRNAs between the control and experimental groups with at least 10000 permutations per analysis. miRs that are significantly different from castrate vehicle treated animals at p≤0.05 were plotted as heatmaps. Two dimensional hierarchical clustering[28] was done using Euclidean Distance and Pearson Correlation; clusters of differentially expressed miRNAs were visualized using heatmaps.

Systems biology analysis of predicted targets of co-expressed miRNAs was performed using our methodology published elsewhere[29]. Briefly, a union of predicted targets for each of co-expressed miRNAs was generated using target prediction algorithm MicroCosm Targets Version 5[30]. A statistical enrichment analysis of functional categories and pathways were performed using Ingenuity Pathway Analysis system IPA 6.0 (Ingenuity Systems, www.ingenuity.com). Functional categories and pathways that are significantly enriched with targets of differentially expressed miRNAs (p≤0.05) were presented as a bar graph. Pathways enriched with miRNA targets were selected for further analysis. Expected changes in expression level of predicted targets were overlaid onto pathway diagram by assigning expression changes proportional to the number of miRNAs that are targeting the same transcripts. An overall effect of changes in expression of miRNAs on expression level of predicted targets was estimated as a sum of increase of expression due to down regulation of corresponding miRNAs and decrease of expression due to down regulation of other miRNAs. Overall effect of changes in expression was visualized using color gradient for overlay of predicted expression changes onto pathway diagrams (Supplemental Figures). These estimates of hypothetical changes of gene expression were used solely for purpose of prioritizing predicted targets and narrowing down list of predictions in order to select those that are most likely to be affected in experimental groups of animals and would be more reliable candidates for validation experiments.

### DICER^−/−^ mice experiments

All animal protocols were approved by Animal Care and Use Committee (University of Tennessee, Memphis) and the experiments were conducted under the approval ID 1763 of University of Tennessee ACUC. MMTV-cre male and DICER flox female mice were obtained from Jax labs (Bar Harbor, Maine) and were bred together to obtain DICER^+/−^ F1 generation. F1 generations were bred together to secure F2 generations. Tail snips were genotyped according to Jax labs protocol and F2 mice that were positive for cre^+/+^ and null for DICER^−/−^ were used in the pharmacodynamic experiments. Tissue specific expression of genes was performed using realtime PCR primers and probes.

## Results

### Androgen responsive tissues express distinct sets of miRs

**Figure 1:**
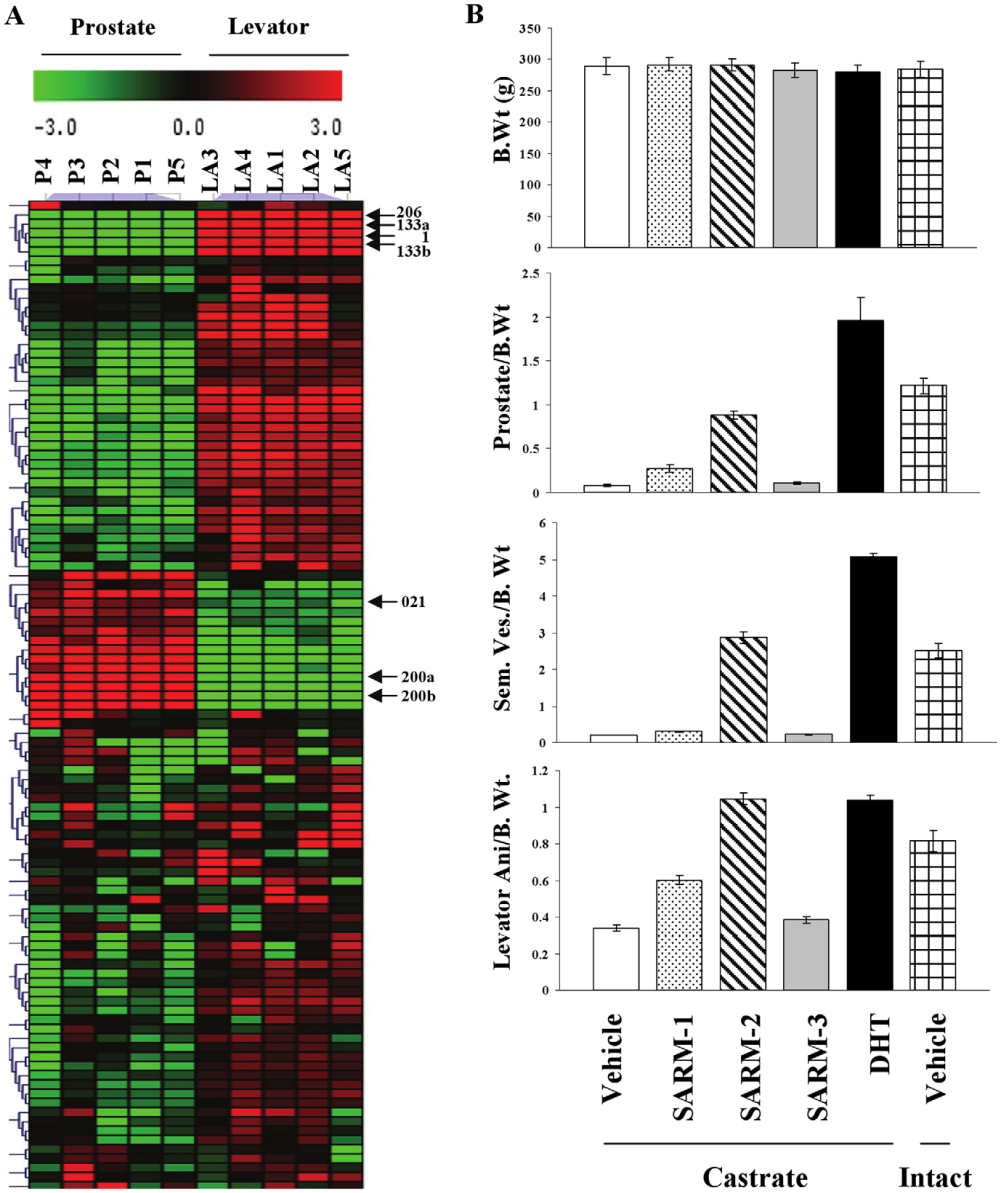
Androgen responsive tissues, prostate and levator ani muscle, express different sets of miRs. A. RNA was extracted from prostate and levator ani muscle of intact Sprague Dawley rats (n = 5; 200 g weight) and the expression of 312 miRs was profiled using realtime PCR. miRs that are statistically different (P<0.05) between the two groups are expressed in the heatmap. P1-P5 = prostate of animals 1–5; LA1-LA5 = levator ani of animals 1–5. B. Pharmacologic effects of AR ligands. Sprague Dawley rats (n = 5; 200 g weight) were castrated and treated subcutaneously for 14 days with vehicle (open bars), 3 mg/day SARM-1 (dotted bars), SARM-2 (hatched bars), an inactive SARM-3 (grey bars) and DHT (black bars). Intact vehicle treated animals are represented by checked bars. At sacrifice, organs were weighed and expressed as raw organ weights. Values are expressed as average ± S.D.

Earlier studies in mice demonstrated that miR expression differs tissue specifically, leading to speculation that differences in miR expression contribute to the characteristic phenotype of these tissues[31]. Androgen-regulated tissues are morphologically and functionally different from one another raising the possibility of distinct miR expression patterns. Since castration of rats leads to regression of androgen-responsive tissues like the prostate and levator ani muscle, changes in the expression of any genes or miRs that occur after castration can be considered to be dependent on circulating androgens[32], [33]. miR profiling in intact rats demonstrated that prostate and levator ani express distinct sets of miRs (Fig. 1A). miRs that are highly expressed in one tissue are not- or weakly- expressed in the other tissue and vice versa. For example, miRs- 206, -133a, -133b, and -1 are expressed robustly in levator ani muscle, but weakly in prostate. On the other hand, miRs-021, -200a, -200b, and -200c are highly expressed in prostate, but not in levator ani muscle. The above mentioned miRs are indicated in Fig. 1A.

Measurement of hormone levels in serum of animals treated with vehicle or SARMs indicated that both SARM-1 and SARM-2 functioned similar to endogenous hormone such as DHT, by not altering the serum testosterone, but by completely inhibiting the castration induced increases in serum luteinizing hormone and follicle stimulating hormone (supplementary table S1). This indicates that these SARMs function directly through AR at the target tissue and not by altering serum hormone levels.

### Androgen treatment up-regulates the expression of a large set of miRs in prostate

To understand the effect of circulating androgens on miR expression in prostate and muscle, Sprague Dawley rats were sham operated and treated with vehicle or castrated and treated subcutaneously for 14 days with vehicle, dihydrotestosterone (DHT), selective androgen receptor modulators, SARM-1 and SARM-2, or an inactive structurally similar, SARM-3. To obtain conclusive results from this study, we utilized AR ligands with varying potency, tissue selectivity and structural diversity[34].

Tissue weights taken at the end of 14 days demonstrated varying levels of androgenic (maintenance of prostate and seminal vesicles weights) and anabolic (maintenance of levator ani muscle weight) effects of these AR ligands (Fig. 1B). While DHT elicited equally potent androgenic and anabolic effects, as evident from the largest increases in prostate, seminal vesicle and levator ani muscle weight, SARM-1 and SARM-2 delivered better anabolic than androgenic effects confirming their tissue selectivity. For example, note that SARM-2 increased levator ani muscle weight to a similar magnitude as DHT, but only restored the prostate to <50% of the size observed in DHT-treated animals. As expected, the inactive SARM-3 had no effect on any tissue.

**Figure 2:**
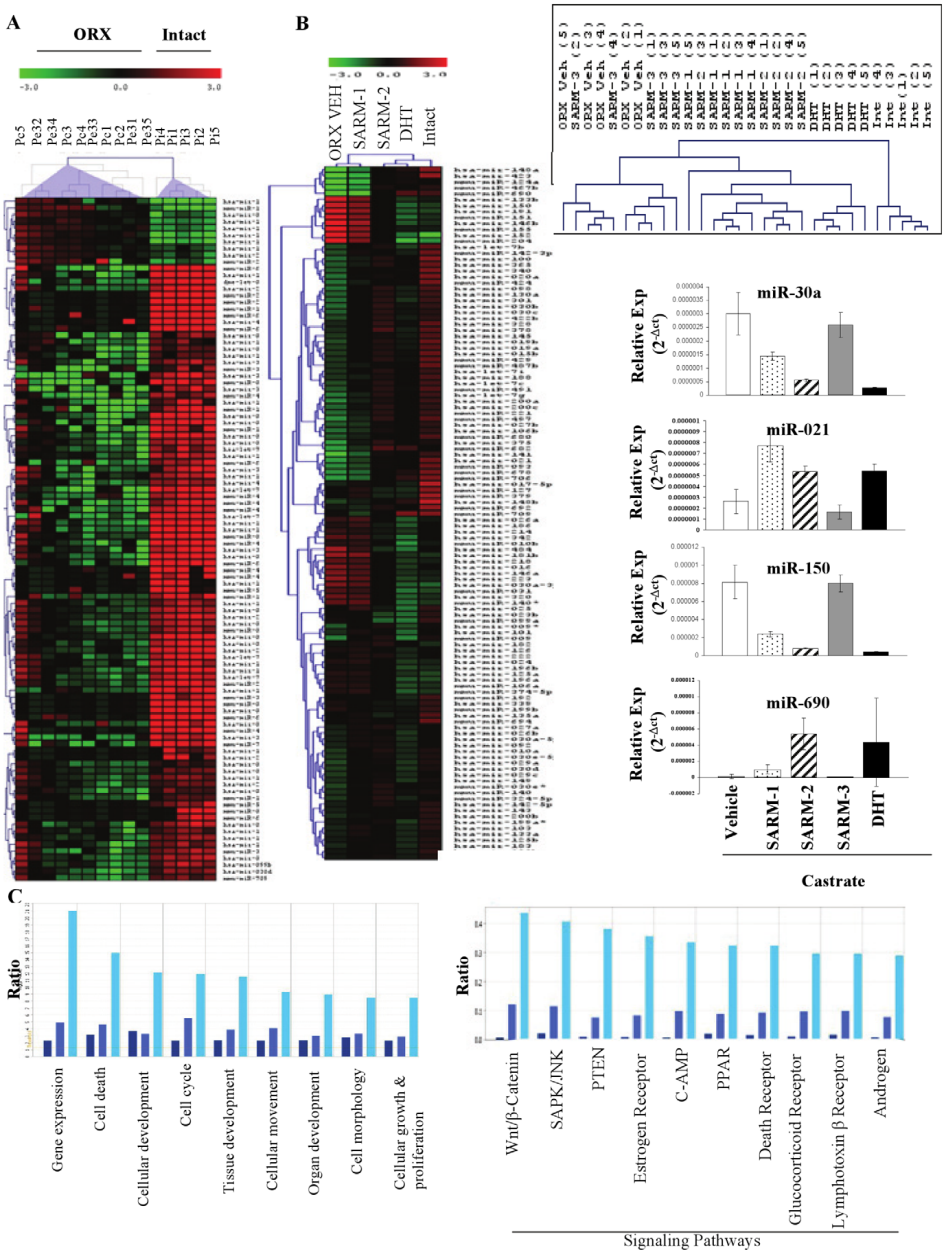
Androgen Receptor ligands alter the expression of miRs in prostate. A. Castration altered miR expression in prostate. RNA was extracted from prostate of rats that were sham operated (Intact) or castrated (ORX) and treated subcutaneously with vehicle for 14 days. miR expression was profiled using realtime PCR and miRs that are statistically different (P<0.05) between the two groups are expressed in the heatmap. (n = 5). B. Treatment of castrated rats with AR ligands altered miR expression in prostate. Sprague Dawley rats (n = 5; 200 g weight) were sham operated and treated subcutaneously with vehicle (Intact) or castrated and treated subcutaneously for 14 days with vehicle (ORX VEH), 3 mg/day SARM-1, SARM-2, an inactive SARM-3 or DHT. RNA from prostate was extracted, expression of 312 miRs was profiled and the miRs that are statistically different (P<0.05) from vehicle-treated castrate animals are expressed in the heatmap. ORX vehicle in the heatmap represents pooled data from vehicle and SARM-3 treated castrate animals. Values are expressed as an average with n = 5. The inset to the right of the heatmap represents the clustering of the individual animals. The numbers in brackets are animal numbers. The bar graphs are representative miR expressions selected from the heatmap. Values are expressed as average ± S.D. (n = 5). C. Ingenuity pathway analyses. The predicted targets of differentially expressed miRs were classified into major functional (left chart) and canonical (right chart) pathways using Ingenuity software. The bars represent pathways corresponding to the miR functional profiles of SARM-1, SARM-2 and DHT treated prostates in that respective order.

Comparison of the miR profiles in prostate of vehicle-treated- sham-operated intact animals versus vehicle-treated- castrated animals (Fig. 2A) showed a striking reduction in a large subset of miRs upon castration, indicating that endogenous androgens maintained the levels of miRs and that expression of miRs was significantly reduced upon interrupted androgen supply. Only a small subset of miRs was up-regulated 14 days after castration.

As castration clearly down-regulated a large subset of miRs in prostate, we examined the effects of androgen treatment of castrated rats to further prove that miR expression was androgen regulated. miR profiling in prostate of rats treated with AR ligands demonstrated that the increase in prostate weight was associated with an increase in miR expression (Fig. 2B). While DHT, which maximally increased prostate weights, elicited the greatest increase in miR expression, partial androgenic SARM-1 elicited only a marginal increase in miR expression in the prostate. On the other hand, SARM-2 elicited a mixed miR profile in the prostate. While many SARM-2 regulated miRs were intermediate in expression between SARM-1 and DHT, some SARM-2 elicited changes were more similar to DHT-treated and sham-operated intact groups. miR expression was maximum in the sham-operated intact animals as compared to the vehicle-treated castrated animals. Not only did the group clustering match perfectly with the androgenic response, but the unsupervised hierarchical clustering of individual animals also matched well with the observed changes in prostate weight (Fig. 2B inset). The inactive SARM-3 failed to alter miR expression indicating the imperative need for AR- binding and -activity to alter miR expression. The right panels of Fig. 2B are representative bar graphs of miRs significantly regulated in prostate. A complete list of regulated miRs (Table S2) and a Venn diagram indicating ligand- dependent distribution of miRs (Figure S1) are depicted in the supplementary figures and tables.

In order to measure the tissue specificity of miRs regulation, expression of miRs was profiled in the liver of rats treated as indicated above. Interestingly, none of miRs were regulated in liver by any of the tested ligands (data not shown), indicating the specificity to androgen responsive tissues such as prostate and levator ani.

Ingenuity pathway analyses to identify affected functional and canonical pathways showed that SARM-1, SARM-2 and DHT activated overlapping but distinct miRs and consequently gene networks, but with varying efficacy (Fig. 2C). The number of genes regulated by each ligand varied tremendously. While SARM-1 regulated the least number of functional and canonical pathway genes, the highly androgenic DHT altered the pathway genes remarkably. Interestingly and similar to changes noted in prostate weight and miRs expression, the number of pathway genes regulated by SARM-2 was intermediate between SARM-1 and DHT regulated genes. The Ingenuity analysis indicated that Wnt signaling pathway is the major canonical pathway affected by the AR ligands in prostate (Figure S2). While DHT activated (red) several genes, SARM-2 predominantly inhibited (green) the genes in this pathway. Validation of the Wnt signaling pathway in the prostate of rats indicated that DHT altered the expression of several genes in this pathway validating the prediction based on the altered miRs (Table S3).

### AR ligands alter expression of miRs in levator ani muscle

**Figure 3:**
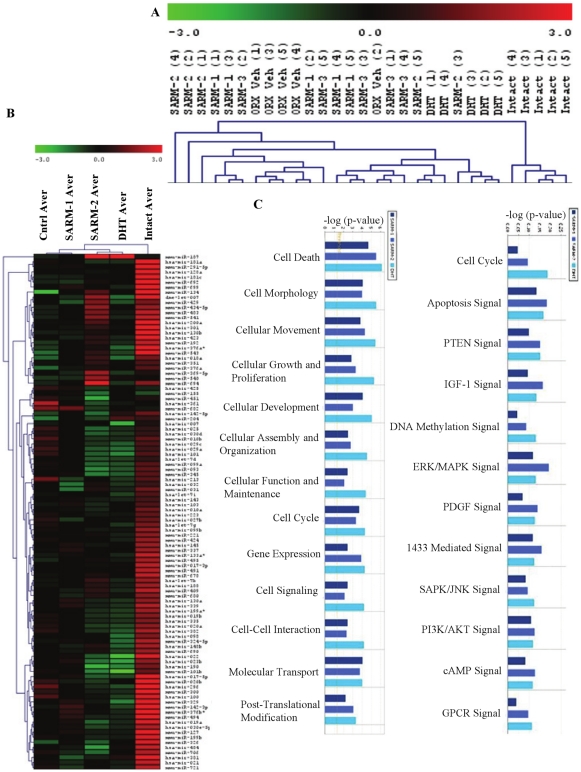
Androgen Receptor ligands alter the expression of miRs in levator ani. A and B. Sprague Dawley rats (n = 5; 200 g weight) were sham operated (Intact) and treated subcutaneously with vehicle or castrated and treated subcutaneously for 14 days with vehicle (ORX VEH (A); Cntrl Aver (B)), 3 mg/day SARM-1, SARM-2, an inactive SARM-3 or DHT. RNA from levator ani was extracted and the expression of 312 miRs were profiled and the miRs that are statistically different (P<0.05) from vehicle treated castrate animals are expressed in the heatmap. Values are expressed as an average with n = 5. (B). Clustering of individual samples are given in panel A. The numbers in bracket are numbers of animals. ORX vehicle in the heatmap represents pooled data from animals treated with vehicle and SARM-3. C. Ingenuity pathway analyses. The predicted targets of differentially expressed miRs were classified into major functional (left chart) and canonical (right chart) pathways using Ingenuity software. The bars represent pathways corresponding to the miR functional profiles of SARM-1, SARM-2 and DHT treated levator ani muscle in that respective order.

Prostate and other secondary sexual organs are bonafide direct target tissues of androgens[35]. Though androgens alter muscle function and composition, this area of research lacks mechanistic characterization and several researchers believe that androgens act through other anabolic pathways like insulin like growth factor-1 to promote muscle growth[36]. We profiled miRs in an androgen-responsive AR expressing muscle, levator ani[37]. Similar to the effect in prostate, SARM-3 failed to alter the expression of any miR compared to vehicle treated-castrate animal controls (Fig. 3A and 3B). Unsupervised hierarchical clustering of individual animals demonstrated that the affected miRs did not correlate well with the anabolic effects on levator ani muscle (Fig. 3A). While intact or sham operated- and DHT- treated animals clustered to the extreme right in the heatmap, SARM-2-, SARM-1- and vehicle- treated castrate animals clustered together indicating that, unlike prostate, no consistent trend was observed (Fig. 3A). However, clustering of the groups correlated well with the anabolic effects, with castrate controls clustered at one end and the sham-operated intact group clustered at the other end of the heatmap (Fig. 3B). Castration significantly reduced most of the miRs in levator ani indicating that, similar to prostate, miR expression in muscle is androgen regulated. However, unlike prostate, treatment with AR ligands failed to produce a consistent pattern of miR regulation in levator ani muscle (Fig. 3B). A list of regulated miRs (Table S4) and a Venn diagram indicating the ligand-dependent distribution of miRs (Figure S3) for muscle are depicted in the supplementary figures and tables.

Ingenuity pathway analyses to identify the functional and canonical pathways showed that the three AR ligands affected similar pathways (Fig. 3C). Unlike the prostate, the number of genes altered by each ligand was almost similar. The major pathway affected by the AR ligands in muscle was the ERK:MAPK signaling pathway (Figure S4), but the genes of this pathway were not consistently regulated by the ligands. DHT and SARM-2 activated or repressed the genes similarly. The lack of separation between DHT and SARM in an anabolic tissue is in concordance with our earlier observations in cell lines[6] and pharmacologic data in rats showing the ability of SARMs to mimic the anabolic effects of DHT in levator ani muscle[38].

### Functional role of miRs in androgen action

Since the AR ligands altered miR expression with near-perfect correlation to andogenicity, we performed *in vitro* and *in vivo* mechanistic studies to determine the functional role of miRs in prostate. Our goal was to determine whether the observed changes in miR expression caused or were the result of the observed changes in tissue size (i.e., were the changes in miR expression imperative for AR function or biomarkers of AR function in these tissues?) (Figure S5).

**Figure 4:**
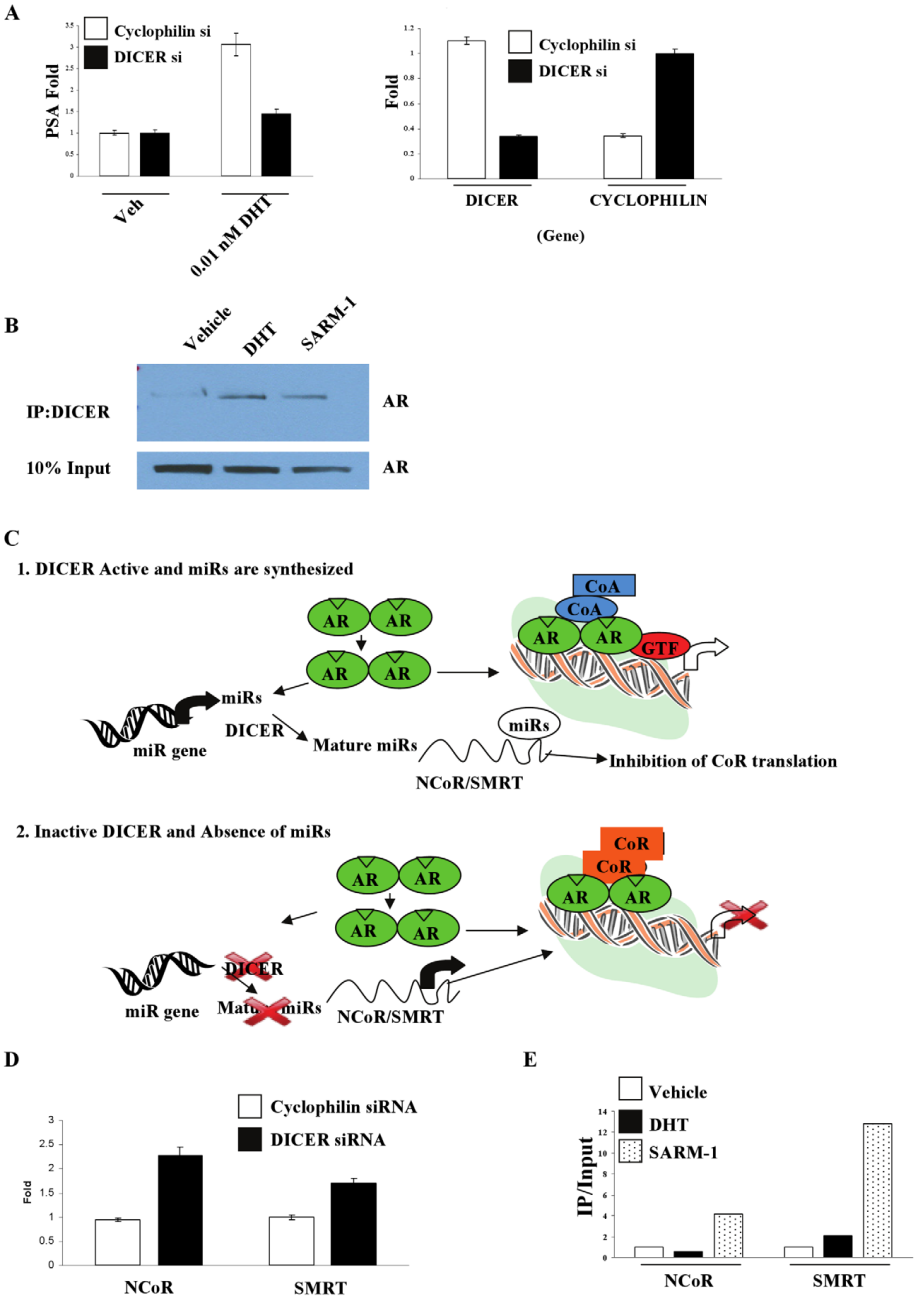
miRs are important for AR function in LNCaP cells. A. DICER siRNA abrogates AR function in LNCaP cells. LNCaP cells were transfected with cyclophilin or DICER siRNA for 6 days and treated with vehicle or 0.01 nM DHT. Expression of PSA (left panel), DICER and cyclophilin (right panel) was measured using realtime PCR. Values are expressed as average ± S.D. (n = 3). B. Ligand dependent interaction of AR with DICER. LNCaP cells were serum starved for 3 days and treated with vehicle, 10 nM DHT or SARM-1 for 6 hrs. Protein was extracted and immunoprecipitated with DICER antibody and western blotted for AR. Representative blot of n = 3 is shown. C. Plausible mechanism for the regulation of AR function by miRs. 1. In the presence of DICER, androgen treatment increases miR- synthesis and –maturation and binding to 3′UTR of corepressors, NCoR and SMRT. This in turn inhibits corepressors translation resulting in augmented AR function. 2. In the absence of DICER, miR-maturation is inhibited leading to enhanced corepressor expression subsequently repressed AR function. D. Inhibition of miR synthesis increases NCoR and SMRT expression. LNCaP cells were transfected with cyclophilin or DICER siRNA for 6 days and the expression of NCoR and SMRT was measured by realtime PCR. Values are expressed as average ± S.D. (n = 3). E. SARM-1- but not DHT-treatment recruits NCoR and SMRT to PSA enhancer. LNCaP cells were serum starved for 3 days and treated with vehicle, 0.1 nM DHT or 10 nM SARM-1 for 2 hrs. Protein was cross linked with DNA and chromatin immunoprecipitation assay (ChIP) was performed using NCoR and SMRT antibodies. Representative of three independent experiments is shown. AR = androgen receptor; CoA = coactivator; CoR = corepressor; GTF = general transcription factor; NCoR = nuclear receptor corepressor; SMRT = silencing mediator of retinoid and thyroid receptors.

To test these hypotheses, the RNAse, DICER, was inhibited in LNCaP prostate cancer cells, using siRNA. DICER, instead of Drosha, was targeted in these studies to understand the importance of pre-miRs, if any, in androgen action. DICER siRNA achieved a more than 70% knockout of DICER compared to the control siRNA (Fig. 4A right panel). Interestingly, inhibition of miR synthesis using DICER siRNA completely abrogated the induction of PSA gene expression by DHT (Fig. 4A left panel).

Since AR and DICER are localized in the cytoplasm and AR shuttles into the nucleus upon ligand binding, interaction between the two proteins was analyzed in the presence or absence of a strong (DHT) or a weak (SARM-1) AR ligand using co-immunoprecipitation studies. Though both proteins were localized in the cytoplasm, there was no interaction between AR and DICER in the absence of ligand[39], [40]. Strikingly the two proteins interacted in the presence of SARM-1 or DHT (Fig. 4B). This indicates that ligand-induced changes in conformation of AR promote its interaction with DICER. Functional consequences of this interaction and the site of this interaction are being analyzed currently.

### Plausible hypothesis and confirmation for the involvement of corepressors

Since miRs inhibit translation or degrade mRNA of a target gene, we hypothesized that the observed inhibition of AR function upon reduced miR synthesis was an indication that the miRs function as inhibitors of AR repressors. Nuclear receptor corepressor (NCoR) and silencing mediator of retinoid and thyroid receptor (SMRT) are two well-characterized repressors of AR function[41]. It is feasible that AR in the presence of ligand increases miR- synthesis and -binding to the 3′UTR of NCoR and SMRT, leading to degradation of the mRNAs for these two corepressors (Fig. 4C1). Degradation of NCoR and SMRT would in turn increase the transcription and translation of AR target genes. On the other hand, in the absence of DICER, miR synthesis and binding to the 3′UTR of corepressors would be impaired, leading to corepressor abundance and AR target gene inhibition (Fig. 4C2).

As a first step to validate this hypothesis, the Sanger miR database was searched to identify the list of miRs that bind to NCoR and SMRT 3′UTRs and compare them to the upregulated miRs in prostate. Consistent with our hypothesis, more than 75% of the NCoR and SMRT binding miRs published in the Sanger database were upregulated in prostate samples of our experiment (Table S5). To confirm experimentally, NCoR and SMRT mRNA levels were measured in LNCaP cells transfected with DICER siRNA. Inhibition of miR synthesis with DICER siRNA led to an increase in transcription of both NCoR and SMRT (Fig. 4D).

Though studies using DICER siRNA confirmed that inhibition of miR maturation increases NCoR and SMRT levels, we used SARM-1 to determine whether a ligand that binds and recruits AR but minimally increases miR expression would promote recruitment. We expected this ligand to increase the recruitment of NCoR and SMRT to an ARE. LNCaP cells were treated with SARM-1, the SARM that minimally altered the miRs, or DHT, a ligand that maximally altered the miRs, and the recruitment of NCoR and SMRT to PSA enhancer was determined using a chromatin immunoprecipitation assay (ChIP assay). Consistent with the hypothesis, treatment of LNCaP cells with SARM-1, but not with DHT, significantly recruited NCoR and SMRT to the PSA enhancer (Fig. 4E), indicating that an AR ligand that minimally increases the miR expression recruited corepressors due to the absence of negative regulation.

### Inhibition of miR maturation leads to androgen insensitivity syndrome

**Figure 5:**
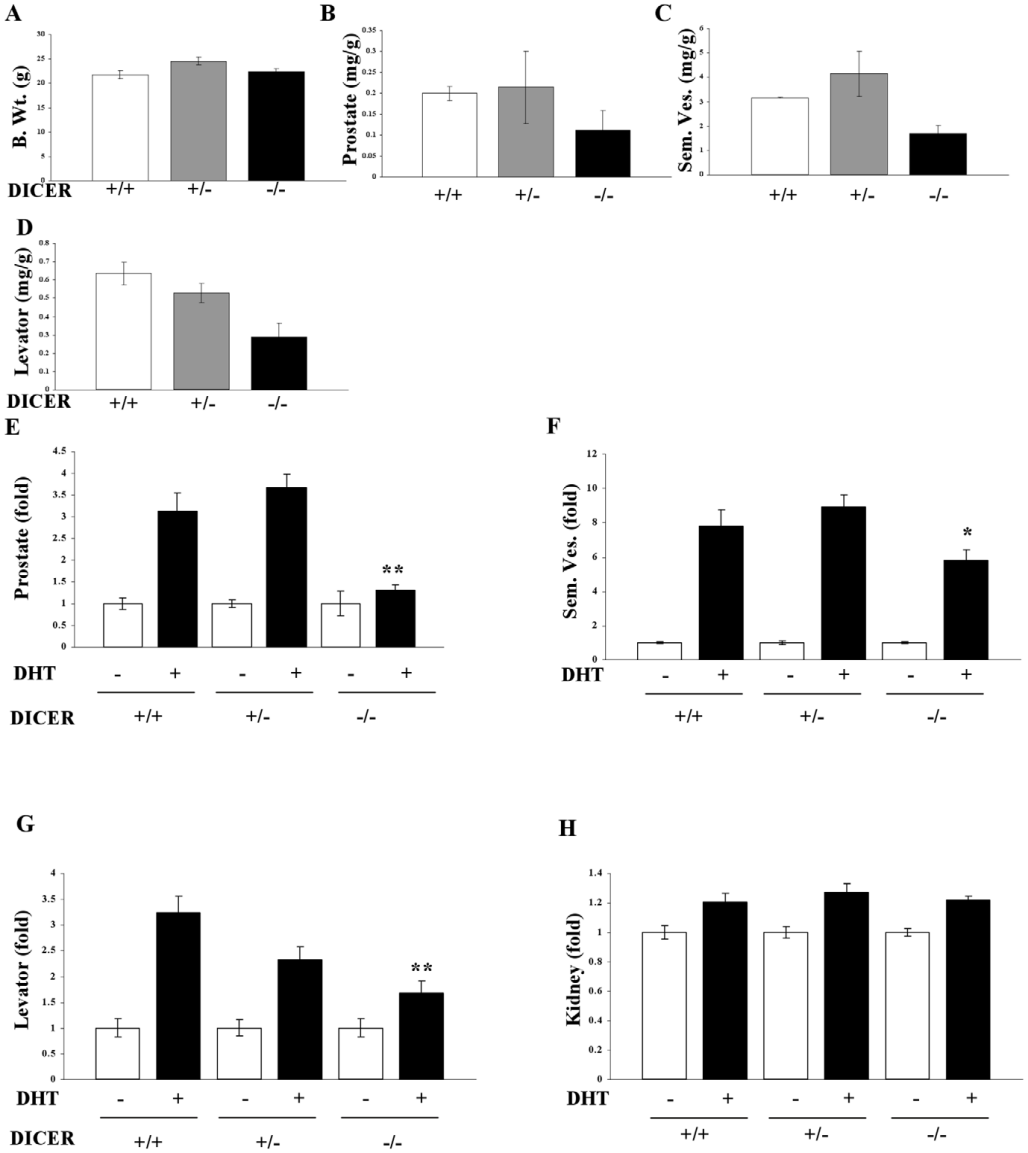
DICER null mice have impaired androgen responsiveness. A. Intact DICER null mice have smaller androgen responsive tissues. MMTV cre and DICER flox mice were crossed to generate tissue selective DICER +/− or DICER −/− mice. Body (A), prostate (B), seminal vesicle (C) and levator ani (D) weights were measured in 6 weeks old F2 generation mice (n = 2). B. Partial responsiveness of DICER homozygous null mice to DHT. Six weeks old wildtype (n = 5), DICER +/− (n = 9) and DICER −/− (n = 5) mice were castrated and treated subcutaneously with vehicle or 10 mg/kg/day DHT for 14 days. The animals were sacrificed and prostate (E), seminal vesicles (F), levator ani (G) and kidney (H) weights were measured and normalized to body weights. Data is expressed as DHT-dependent fold change from the respective vehicle groups. **-significant at P<0.01 and *-significant at P<0.05 from DHT treated wildtype animals.

**Figure 6:**
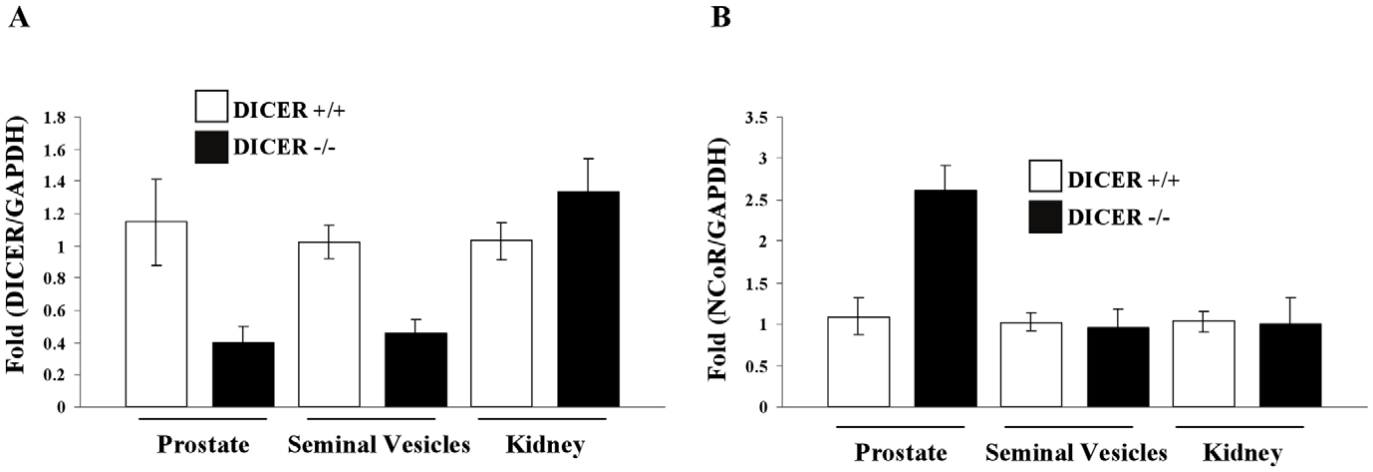
**A.** Expression of DICER in wildtype and DICER −/− mice in prostate, seminal vesicles and kidney from animals described under Fig. 5E–5G. White bars are wildtype mice and black bars are DICER −/− mice. **B.** Expression of NCoR and SMRT in wildtype and DICER −/− mice in prostate, seminal vesicles and kidney from animals described under Fig. 5E–5G. White bars are wildtype mice and black bars are DICER −/− mice.

Since the results in LNCaP cells strongly suggested the importance of miRs for AR function, tissue-specific DICER knockout animals were generated by crossing MMTV-cre mice and DICER-flox mice. Earlier studies indicated that crossing any flox-gene with MMTV-cre inhibits the gene expression in seminal vesicles, prostate, breast and other hormone-dependent tissues[42], [43]. Mice were genotyped using a PCR-based approach to identify the DICER^+/−^ and DICER^−/−^ mice. When these mice attained 6 weeks of age, they were sacrificed and weights of prostate, levator ani and seminal vesicles were measured. No change in the body weight was observed in these mice (Fig. 5A). However, the androgen-dependent tissues such as prostate, seminal vesicles and levator ani muscle were much smaller in the DICER^−/−^ mice compared to the wildtype or DICER^+/−^ mice (Fig. 5B–D). Since this phenotype correlates with androgen insensitivity syndrome, the response of castrated mice to androgen administration was evaluated. Six week old wildtype, DICER^+/−^ and DICER^−/−^ mice were castrated and treated with vehicle or 10 mg/kg/day DHT subcutaneously for 14 days and the weights of prostate, seminal vesicles, levator ani and androgen independent kidneys were evaluated. Similar to the intact mice, wildtype and DICER^+/−^ mice responded to DHT treatment with increase in the weights of prostate, seminal vesicles and levator ani muscle (Fig. 5E–H). However, DICER^−/−^ mice failed to respond to DHT treatment with little to no increase in prostate and levator ani weights and only a partial response in seminal vesicles weight gain. The weights of an androgen-independent tissue, kidneys, were comparable in all the three genotypes (Fig. 5H). Tissue specific knockout of DICER in prostate and seminal vesicles, but not in kidney, of DICER^−/−^ mice was confirmed using realtime PCR (Fig. 6A).

Based on our hypothesis and LNCaP results, NCoR levels were measured in prostate, seminal vesicles and kidneys. As demonstrated using DICER siRNA in LNCaP cells, NCoR expression was significantly higher in DICER^−/−^ mice compared to wildtype mice in prostate but not in kidneys (Fig. 6B). Interestingly, NCoR levels were not altered in seminal vesicles, perhaps indicating prostate specificity in its regulation.

### Detection of miRs in serum miR-214 and miR-125a

Multiple age-related disorders occur due to androgen deficiency, suggesting that androgen supplementation is useful to overcome these disorders. However, one of the major concerns related to androgen therapy is an increase in prostate proliferation and potential effects on benign prostatic hyperplasia or subclinical prostate cancer. To determine whether we could identify any miR in serum that could correlate to androgen action in prostate, serum miRs were profiled in castrated rats that were administered with SARM-1, SARM-2 and DHT as indicated above in fig. 1, 2, 3. All the three AR ligands increased the expression of various miRs in serum (Table S6). However, only two miRs, miR-214 and miR-125a, positively correlated with androgen action in prostate. Serum concentrations of both these miRs were increased by SARM-2 and DHT, but not by SARM-1 or SARM-3. In addition, these miRs were altered by SARM-2 and DHT in prostate. This observation requires validation using larger sample sets. Studies to examine the dose- and time- dependent regulation of miR-214 and miR-125a, by androgens, are ongoing in our laboratories.

## Discussion

miR research is predominantly focused in oncology and little research has been performed to understand the importance of miRs in normal tissue development. Moreover, the significance of miRs in hormone-action or steroid receptor-function is an emerging field of research. The classical view of hormone action is one that depicts ligand-bound AR being recruited to the promoter of target genes to mediate the transcription and translation process. The results presented in this manuscript demonstrate that miRs are also mediators of androgen action. Instead of the classical one-step model of gene activation, AR also appears to regulate gene expression through a three-step pathway including miR activation, corepressor suppression and DNA interaction to elicit its action.

Though the work clearly demonstrates that androgens increase the expression of a large set of miRs, it is possible that only a few miRs may be mediating androgen action in prostate. miR-21 and miR-125, which have been published to be androgen responsive and play a role in prostate carcinogenesis, are also upregulated in normal prostate[22], [23]. Our dataset indicates that miR-21 is highly responsive to androgen treatment in normal prostate. Even a weakly androgenic AR ligand, SARM-1, was able to induce the expression of miR-21 in prostate. As miR-21 was not increased in levator ani muscle, and unregulated by the inactive SARM-3, this could also function as a prostate-specific marker of androgen action.

Despite these results, several questions remain unanswered. The role of Drosha, intracellular localization of DICER, whether loss of AR responsiveness is due to increased NCoR/SMRT expression and binding of the upregulated miRs to NCoR and SMRT 3′UTRs are some worth mentioning. The most interesting question is the reasons for AR to only ligand-dependently interact with DICER during its nuclear translocation process. Since the coimmunoprecipitation experiments were performed six hours after ligand treatment, the time at which nuclear localization of AR is complete, it raises the possibility that DICER completes its miR processing quickly before translocating to the nucleus. Moreover, the lack of interaction between unliganded AR and DICER, despite their co-existence in the cytoplasm, suggests that conformational changes in AR lead to its interaction with DICER. Since the interaction took place with two different classes of AR ligands which are capable of inducing diverse structural modifications, we speculate that only events common to both conformations could be responsible for this interaction[44].

The cause and effect relationship between cellular proliferation and miR expression (supplementary figure S5), whether androgens activate miRs to proliferate the tissues or *vice versa*, was clarified by the results obtained with DHT in DICER −/− knockout mice. Though DHT increased the size of prostate and levator ani comparable to the intact animals, it was unable to restore the miR expression to that of the intact animals. This indicates the discordance between androgen-regulated cell numbers and miR expression as even partial restoration of miR expression was sufficient to increase the tissue size. Similarly, prostate and levator ani in DICER −/− mice did not respond to DHT treatment, indicating that miRs mediate androgen actions to subsequently increase cellular proliferation.

Interestingly, DICER knockdown failed to increase NCoR expression in seminal vesicles. This could potentially be due to the lack of complete regulation of DHT action in DICER −/−. This also indicates the possible tissue selective regulation of androgen action by miRs.

A few seminal studies have been performed to understand the role of miRs in ER biology. Similar to androgens, estrogens also activate miRs during female reproductive development. Knockout of DICER in uterus and ovaries rendered these mice infertile demonstrating the importance of miRs in female reproductive development[45], [46]. The major difference between estrogen and androgen action is that estrogens work at Drosha level, whereas androgens appear to work at the DICER level, indicating that even presence of pre-miRs are insufficient for androgen action. Also, the DICER^+/−^ data, similar to other publications, indicates that presence of one allele is sufficient to rescue the phenotype of these mice[47].

Collectively, these results explained a mechanism for androgen action in prostate and muscle and found a tight relationship between AR, miRs and corepressors. These studies also suggest that miRs are indirect RNA activators of AR.

## Supporting Information

Table S1 Hormone levels were measured in serum of animals castrated and treated with vehicle, SARM-1 or SARM-2 or intact animals treated with vehicle. Values are expressed as mean ± S.D (n = 5).
(0.05 MB TIF)

Table S2 Statistically significant miRs in prostate in groups treated with vehicle or AR ligands as detailed in Fig. 2.
(0.02 MB PDF)

Table S3 Validation of Wnt-β-catenin pathway genes in prostate of DHT or SARM-1 treated animals. RNA from prostate (n = 5) of vehicle- or DHT- treated castrate animals that were used for miR profiling represented in Fig. 2 were reverse transcribed and the expression of Wnt-β-catenin pathway genes measured using PCR array (SA Biosciences, Frederick, MD). Statistically different genes in DHT- treated groups compared to vehicle- treated group are expressed.
(0.06 MB TIF)

Table S4 Statistically significant miRs in levator ani in groups treated with vehicle or AR ligands as detailed in Fig. 3.
(0.08 MB TIF)

Table S5 miRs interacting with the 3' UTR of NCoR and SMRT (Sanger database) that are up-regulated by AR ligands in prostate (Fig. 2).
(0.06 MB TIF)

Table S6 Expression of miRs in serum. RNA was extracted using Qiagen RNA extraction kits from 1 ml serum from animals that were treated as indicated under Fig. 2. The expression of 312 miRs was profiled using realtime PCR based methods.
(0.07 MB TIF)

Figure S1 Venn diagram of the statistically significant miRs in prostate. miRs that are significantly altered by the treatments described in Fig. 2 are categorized into Venn diagram.
(0.06 MB TIF)

Figure S2 Wnt-β-catenin pathway. Genes predicted to be targets of microRNAs regulated by DHT and SARM-2 in prostate. Genes marked in red are predicted to be up-regulated, genes marked in green are predicted to be down-regulated and genes marked in white are predicted to be non-regulated as a result of microRNA regulation by the respective ligands (see supplementary statistical methods section for details).
(0.21 MB TIF)

Figure S3 Venn diagram of statistically significant miRs in levator ani. miRs that are significantly altered by the treatments described in Fig. 3 are categorized into Venn diagram.
(0.05 MB TIF)

Figure S4 ERK-MAPK pathway. Genes predicted to be targets of microRNAs regulated by DHT and SARM-1 in levator ani muscle. Genes marked in red are predicted to be up-regulated, genes marked in green are predicted to be down-regulated and genes marked in white are predicted to be non-regulated as a result of microRNA regulation by the respective ligands (see statistical methods section for details).
(0.23 MB TIF)

Figure S5 Possible predicted mechanism for the regulation of miRs by AR ligands.
(0.08 MB TIF)
