# Supplementary figures and images for: MicroRNAs Are Mediators of Androgen Action in Prostate and Muscle
Source: PLoS One. 2010 Oct 27;5(10):e13637. doi: 10.1371/journal.pone.0013637 (PMC2965097; doi:10.1371/journal.pone.0013637)

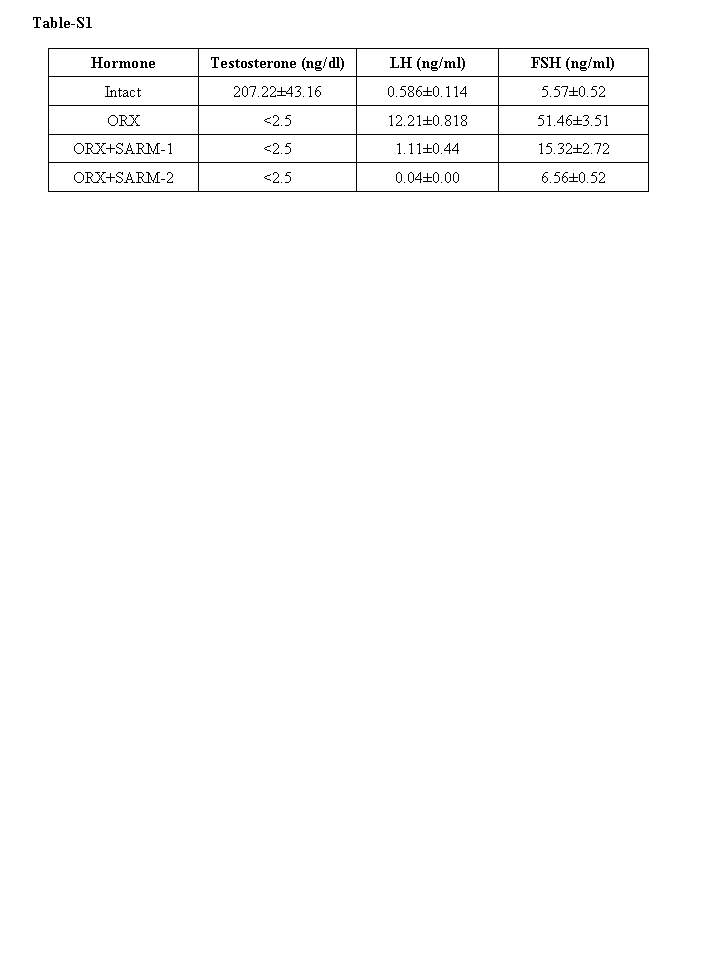

Supplement: Table S1 — Hormone levels were measured in serum of animals castrated and treated with vehicle, SARM-1 or SARM-2 or intact animals treated with vehicle. Values are expressed as mean ± S.D (n = 5). (0.05 MB TIF) [file pone.0013637.s001.tif]

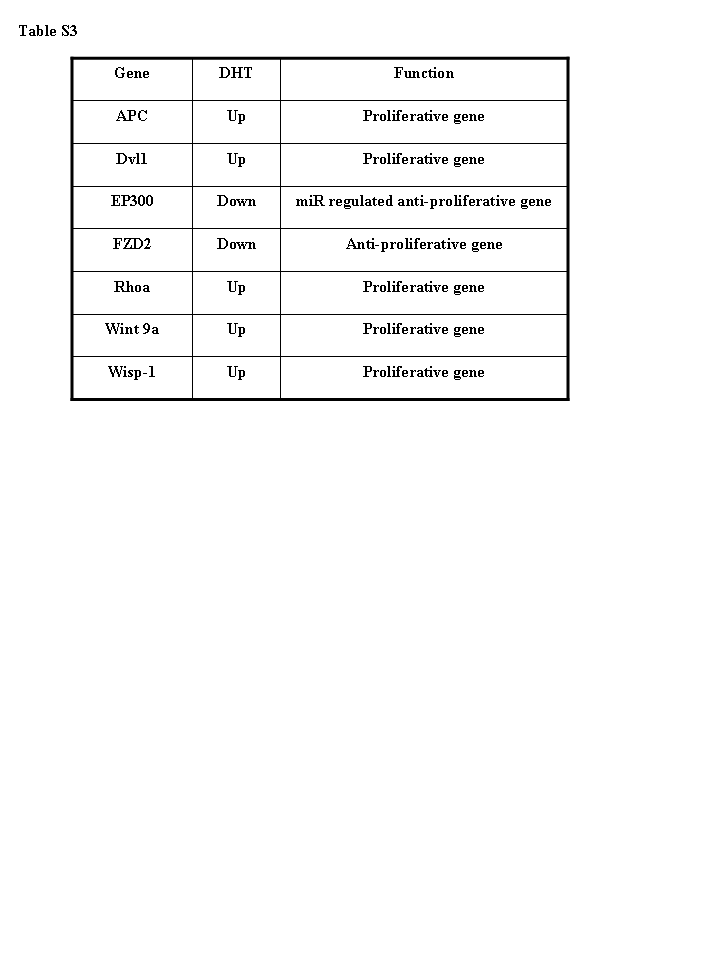

Supplement: Table S3 — Validation of Wnt-β-catenin pathway genes in prostate of DHT or SARM-1 treated animals. RNA from prostate (n = 5) of vehicle- or DHT- treated castrate animals that were used for miR profiling represented in Fig. 2 were reverse transcribed and the expression of Wnt-β-catenin pathway genes measured using PCR array (SA Biosciences, Frederick, MD). Statistically different genes in DHT- treated groups compared to vehicle- treated group are expressed. (0.06 MB TIF) [file pone.0013637.s003.tif]

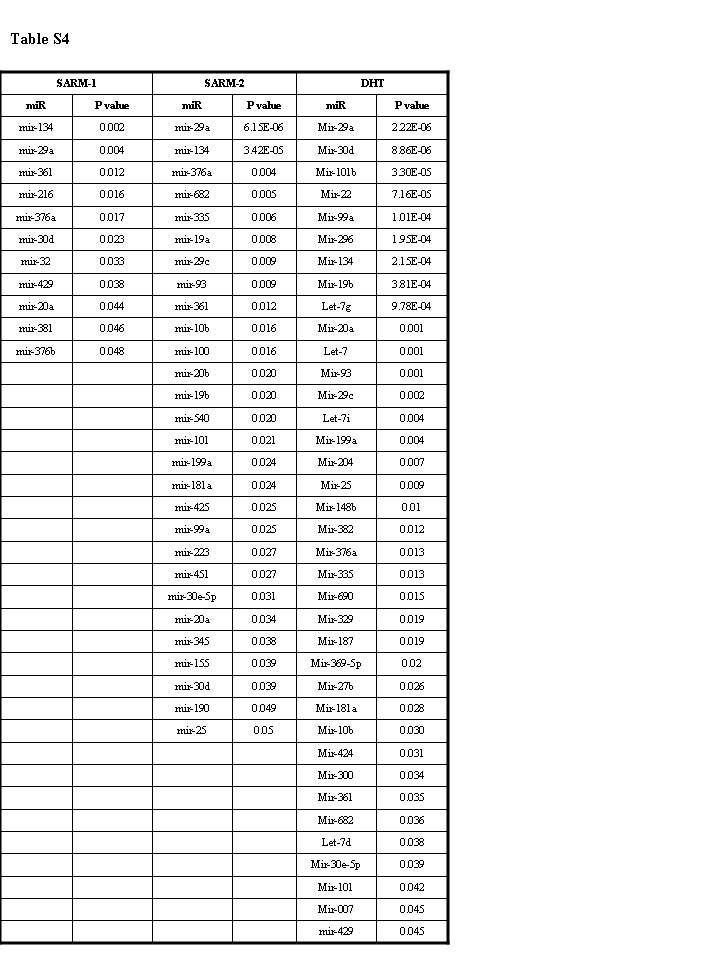

Supplement: Table S4 — Statistically significant miRs in levator ani in groups treated with vehicle or AR ligands as detailed in Fig. 3. (0.08 MB TIF) [file pone.0013637.s004.tif]

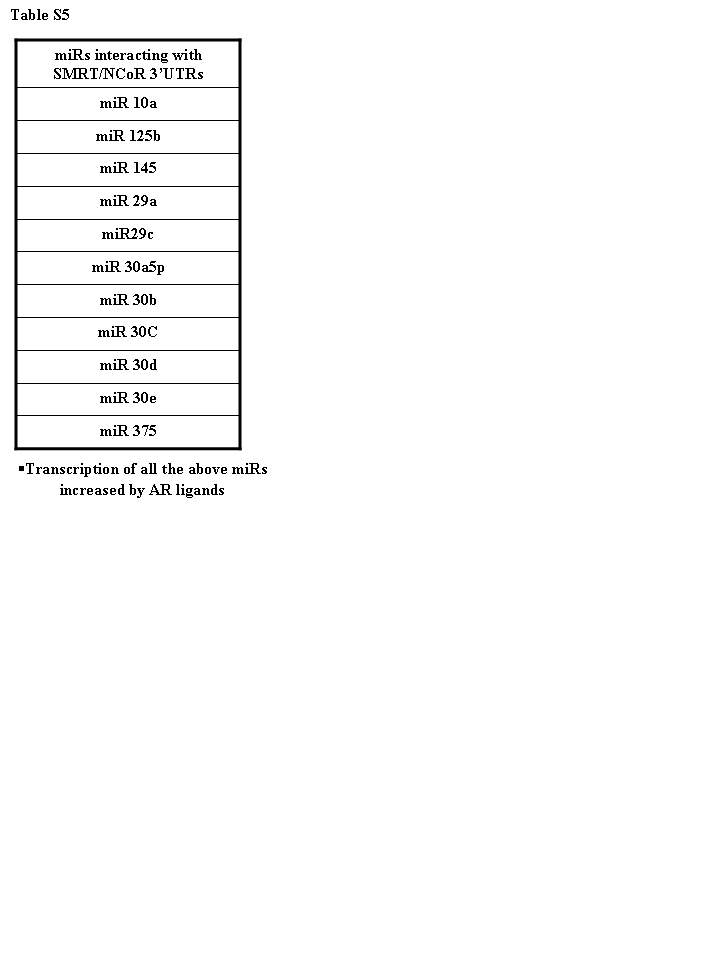

Supplement: Table S5 — miRs interacting with the 3' UTR of NCoR and SMRT (Sanger database) that are up-regulated by AR ligands in prostate (Fig. 2). (0.06 MB TIF) [file pone.0013637.s005.tif]

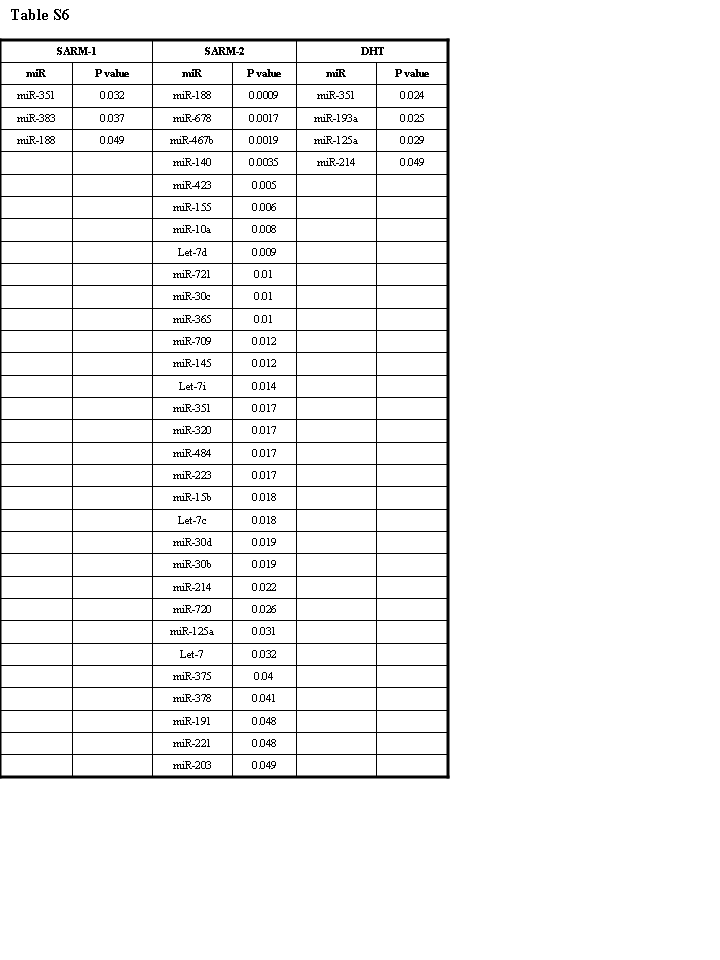

Supplement: Table S6 — Expression of miRs in serum. RNA was extracted using Qiagen RNA extraction kits from 1 ml serum from animals that were treated as indicated under Fig. 2. The expression of 312 miRs was profiled using realtime PCR based methods. (0.07 MB TIF) [file pone.0013637.s006.tif]

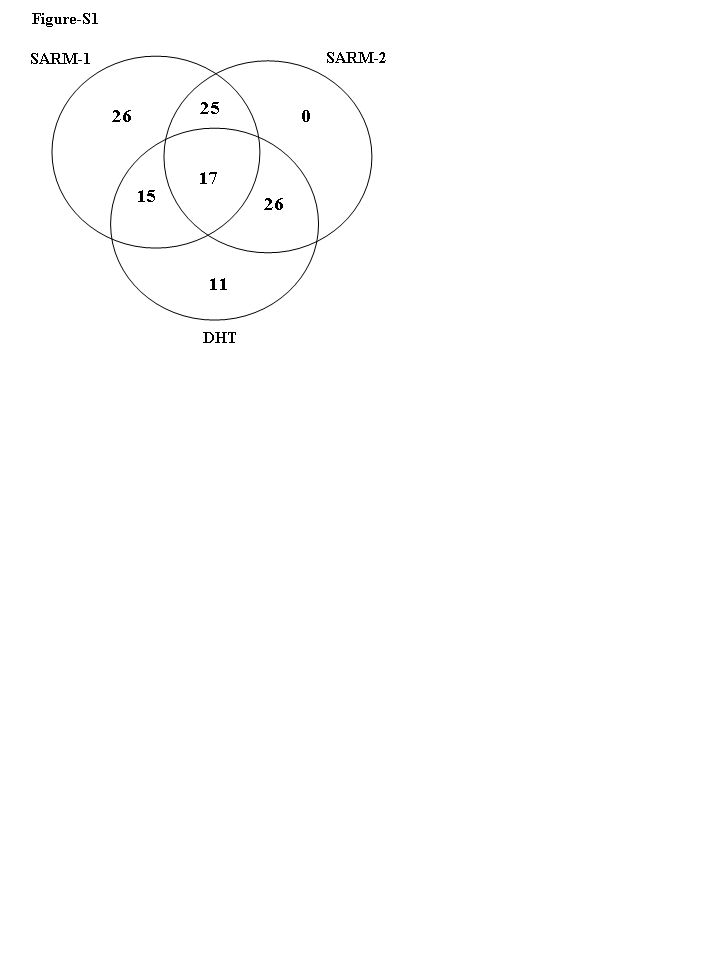

Supplement: Figure S1 — Venn diagram of the statistically significant miRs in prostate. miRs that are significantly altered by the treatments described in Fig. 2 are categorized into Venn diagram. (0.06 MB TIF) [file pone.0013637.s007.tif]

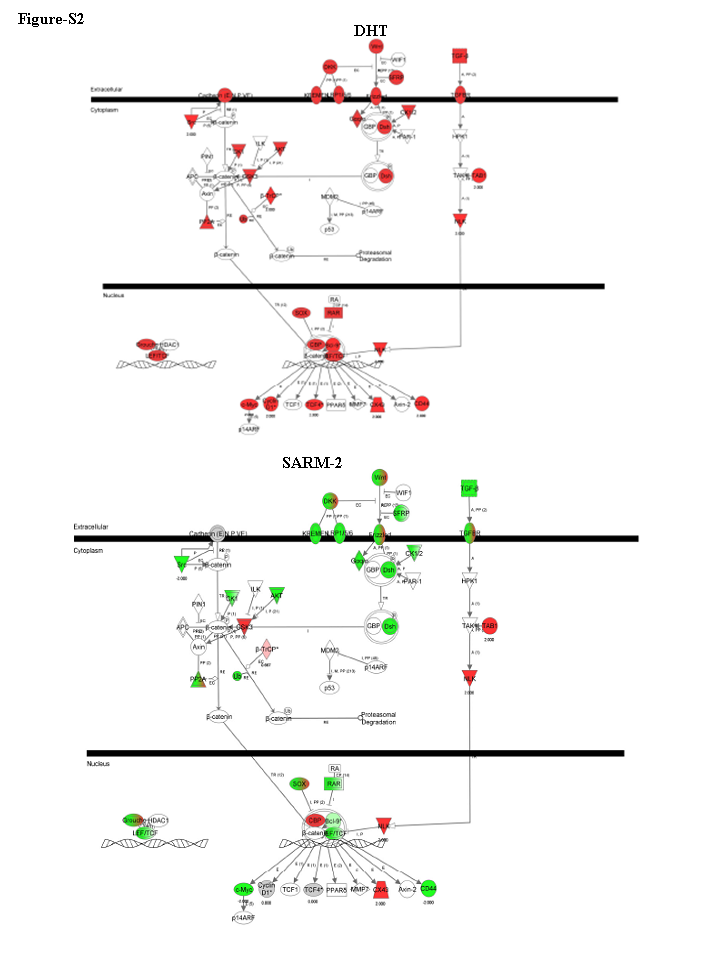

Supplement: Figure S2 — Wnt-β-catenin pathway. Genes predicted to be targets of microRNAs regulated by DHT and SARM-2 in prostate. Genes marked in red are predicted to be up-regulated, genes marked in green are predicted to be down-regulated and genes marked in white are predicted to be non-regulated as a result of microRNA regulation by the respective ligands (see supplementary statistical methods section for details). (0.21 MB TIF) [file pone.0013637.s008.tif]

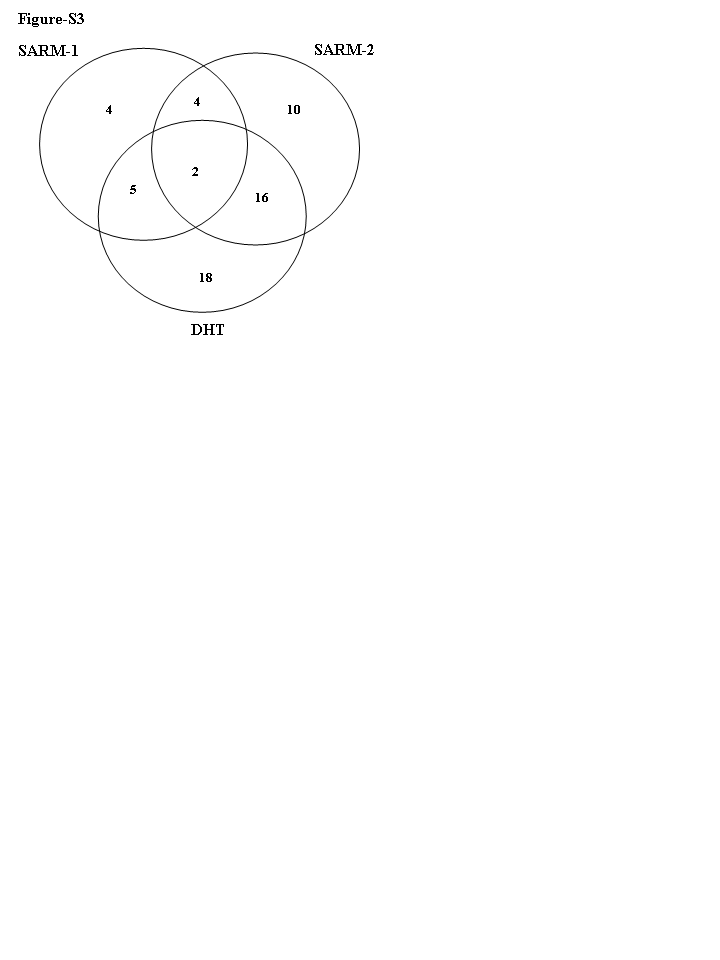

Supplement: Figure S3 — Venn diagram of statistically significant miRs in levator ani. miRs that are significantly altered by the treatments described in Fig. 3 are categorized into Venn diagram. (0.05 MB TIF) [file pone.0013637.s009.tif]

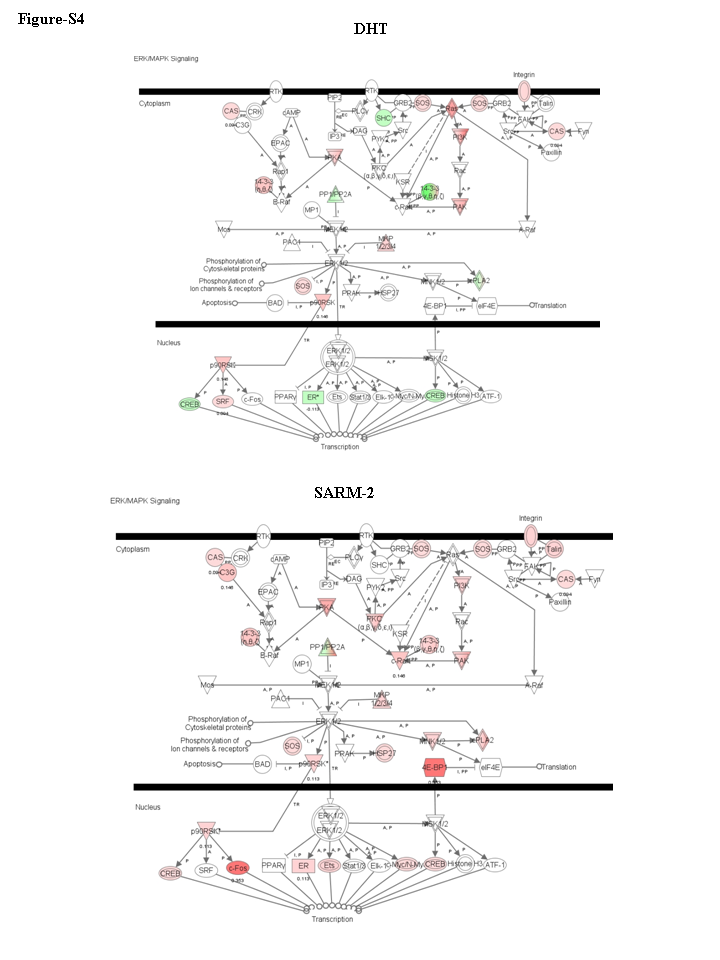

Supplement: Figure S4 — ERK-MAPK pathway. Genes predicted to be targets of microRNAs regulated by DHT and SARM-1 in levator ani muscle. Genes marked in red are predicted to be up-regulated, genes marked in green are predicted to be down-regulated and genes marked in white are predicted to be non-regulated as a result of microRNA regulation by the respective ligands (see statistical methods section for details). (0.23 MB TIF) [file pone.0013637.s010.tif]

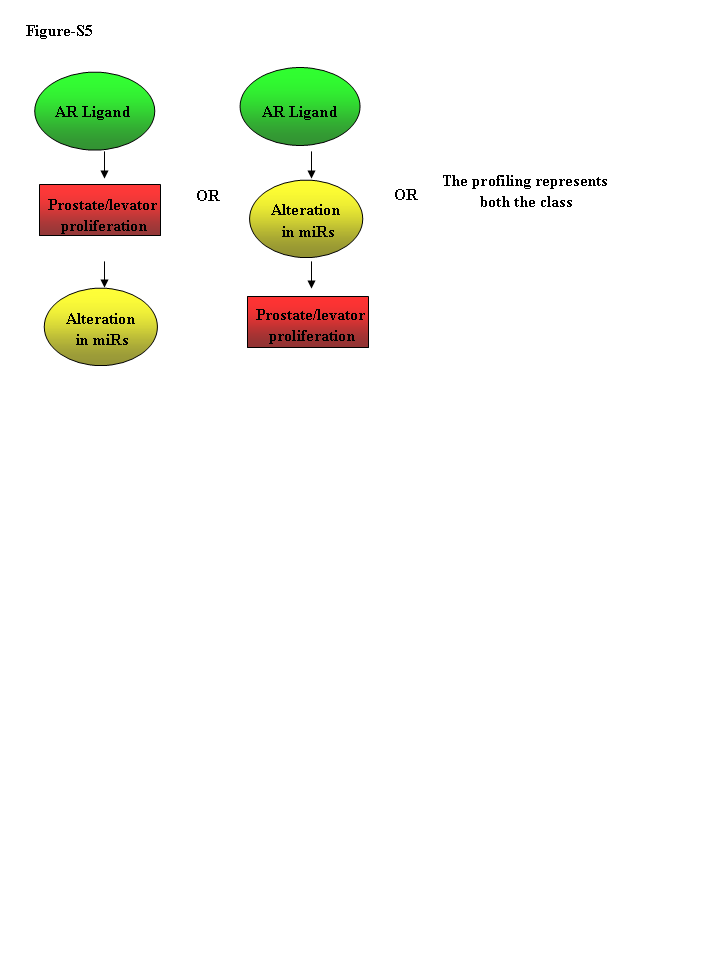

Supplement: Figure S5 — Possible predicted mechanism for the regulation of miRs by AR ligands. (0.08 MB TIF) [file pone.0013637.s011.tif]
