# Supplementary material for: MicroRNAs Are Mediators of Androgen Action in Prostate and Muscle
Source: PLoS One. 2010 Oct 27;5(10):e13637. doi: 10.1371/journal.pone.0013637 (PMC2965097; doi:10.1371/journal.pone.0013637)
Supplement: Table S2 — Statistically significant miRs in prostate in groups treated with vehicle or AR ligands as detailed in Fig. 2. (0.02 MB PDF) [file pone.0013637.s002.pdf]

Table S2

SARM-1		SARM-2		DHT		Intact	
miR	P value	miR	P value	miR	P value	miR	P value
Let-7b	0.012	Let-7c	0.069	Let-7g	0.025	Let-7b	5.78E-06
Let-7c	0.003	Let-7g	6.57E-04	mir-016	1.35E-06	Let-7c	1.97E-05
Let-7g	6.19E-04	Let-7i	0.012	mir-021	0.002	Let-7g	9.34E-06
Let-7i	0.003	mir-10a	0.037	mir-024	1.88E-04	Let-7i	7.51E-05
mir-009*	0.012	Mir-15b	0.005	mir-026a	1.01E-04	mir-009	1.45E-04
mir-010a	0.003	mir-16	2.16E-05	mir-30a-3p	1.74E-08	mir-10a	3.87E-05
mir-15b	8.58E-05	mir-19b	0.017	mir-30a-5p	0.018	mir-15b	1.97E-07
mir-16	0.015	mir-21	0.001	mir-30b	0.021	mir-17-5p	1.90E-05
mir-19a	0.029	mir-24	0.004	mir-125a	6.09E-04	mir-19a	3.22E-05
mir-19b	0.001	mir-26a	0.0133	mir-126	0.001	mir-19b	1.62E-05
mir-20a	0.013	mir-30a-3p	5.88E-07	mir-133b	0.001	mir-20a	2.58E-05
mir-21	0.002	mir-30b	5.89E-04	mir-141	6.29E-05	mir-21	4.82E-07
mir-26b	0.002	mir-30c	0.004	mir-146a	1.01E-06	mir-23b	0.014
mir-27a	9.08E-05	mir-100	0.005	mir-146b	6.11E-08	mir-25	0.001
mir-29a	0.013	mir-106b	0.033	mir-148a	2.44E-06	mir-26b	0.001
Mir-29c	0.007	mir-125a	0.037	mir-148b	0.015	mir-27a	0.024
mir-30a-3p	0.011	mir-126	0.048	mir-150	4.24E-07	mir-27b	0.021
mir-30a-5p	6.59E-05	mir-133b	3.29E-10	mir-152	9.05E-06	mir-29a	3.22E-06
mir-30b	5.04E-05	mir-141	3.21E-06	mir-181b	8.49E-05	mir-29c	8.73E-04
mir-30c	0.012	mir-146a	6.23E-06	mir-186	0.011	mir-30a-3p	0.0083
mir-30d	0.045	mir-146b	5.06E-08	mir-191	9.28E-11	mir-30a-5p	0.0012
mir-30e-5p	0.018	mir-148a	3.70E-06	mir-195	0.011	mir-30b	1.52E-07
mir-92	2.73E-05	mir-150	1.28E-09	mir-196a	2.75E-04	mir-30c	1.32E-05
mir-98	0.003	mir-152	2.32E-04	mir-196b	4.24E-04	mir-30d	8.97E-05
mir-100	0.019	mir-181b	0.004	mir-199a	0.009	mir-30e-5p	7.28E-04
mir-101	0.026	mir-188	0.036	mir-200a	3.22E-04	mir-92	0.04
mir-106b	0.039	mir-191	3.88E-11	mir-200c	0.004	mir-98	7.14E-04
mir-130a	0.021	mir-200a	2.04E-05	mir-214	0.002	mir-99b	2.25E-04
mir-133b	1.49E-05	mir-200c	1.64E-04	mir-218	1E-05	mir-100	7.04E-07
mir-141	0.029	mir-214	0.019	mir-222	0.037	mir-101	0.007
mir-145	0.033	mir-218	1.56E-05	mir-223	3.94E-08	mir-103	0.009
mir-146a	0.008	mir-223	3.15E-05	mir-320	3.61E-05	mir-106b	0.001
mir-146b	6.68E-06	mir-301	0.017	mir-331	0.016	mir-125b	1.23E-04
mir-148a	3.43E-04	mir-320	8.18E-05	mir-339	0.023	mir-130a	0.004
mir-149	9.75E-06	mir-342	0.011	mir-342	0.003	mir-133a	0.002
mir-150	1.42E-04	mir-375	0.004	mir-375	0.019	mir-133b	7.15E-08
mir-152	0.017	mir-423	6.52E-06	mir-423	3.33E-04	mir-135a	0.035

Table S2 (Cont…)

SARM-1		SARM-2		DHT		Intact	
**miR**	**P value**	**miR**	**P value**	**miR**	**P value**	**miR**	**P value**
Mir-181b	0.032	mir-484	8.28E-05	mir-484	0.005	mir-141	9.11E-08
Mir-182	0.017	mir-10b	0.03	mir-10b	2.67E-04	mir-142-5p	0.006
Mir-183	0.018	mir-031	4.58E-04	mir-31	8.28E-07	mir-143	0.005
Mir-186	0.006	mir-093	2.16E-04	mir-93	0.003	mir-145	6.06E-06
Mir-191	1.89E-05	mir-124a	6.05E-05	mir-99a	0.033	mir-146a	0.002
Mir-200a	2.24E-04	mir-140	2.82E-05	mir-106a	2.79E-04	mir-146b	1.32E-05
Mir-200c	7.56E-06	mir-151	4.95E-04	mir-124a	5.76E-06	mir-148a	1.94E-08
Mir-222	0.037	mir-155	9.96E-07	mir-140	2.61E-05	mir-148b	2.22E-05
Mir-223	4.81E-04	mir-204	0.004	mir-151	6.50E-06	mir-149	2.57E-04
Mir-301	3.50E-04	mir-221	3.85E-05	mir-155	7.12E-05	mir-150	2.40E-06
Mir-320	0.049	mir-324-5p	0.007	mir-199b	0.041	mir-152	0.003
Mir-328	0.037	mir-374-5p	0.001	mir-204	7.05E-04	mir-183	6.20E-06
Mir-365	6.08E-04	mir-467b	1.11E-05	mir-374-5p	2.14E-06	mir-188	3.86E-06
Mir-375	8.27E-04	mir-497	0.015	mir-424	0.028	mir-191	1.49E-06
Mir-378	0.02	mir-678	6.32E-05	mir-467b	4.91E-05	mir-199a	6.11E-05
Mir-422b	0.002	mir-682	0.020	mir-542-5p	0.027	mir-200a	9.42E-09
Mir-423	1.29E-05	mir-690	6.09E-06	mir-678	0.002	mir-200b	2.47E-04
Mir-484	1.91E-04	mir-692	0.005	mir-682	7.57E-04	mir-200c	6.41E-07
Mir-009	7.66E-04	mir-706	4.84E-04	mir-690	1.92E-05	mir-222	0.024
Mir-30e*	0.004	mir-709	1.32E-04	mir-706	0.003	mir-223	0.005
Mir-093	8.64E-04			mir-709	0.007	mir-301	5.35E-05
Mir-124a	4.90E-05			mir-720	0.011	mir-320	0.024
Mir-140	0.003					mir-328	0.003
Mir-155	1.17E-05					mir-339	0.021
Mir-199b	9.99E-04					mir-340	1.45E-05
Mir-204	0.029					mir-365	9.00E-06
Mir-221	5.42E-05					mir-375	0.002
Mir-424	0.032					mir-378	3.60E-04
Mir-467b	2.51E-05					mir-422b	2.73E-04
Mir-497	9.54E-04					mir-423	1.80E-09
Mir-678	0.020					mir-009	3.82E-05
Mir-680	0.0159					mir-093	1.29E-07
Mir-682	7.14E-05					mir-099a	6.12E-05
Mir-690	1.18E-04					mir-124a	0.001
						mir-127	1.96E-04
						mir-140	0.004
						mir-142-3p	6.17E-04

Table S2 (Cont…)

SARM-1		SARM-2		DHT		Intact	
**miR**	**P value**	**miR**	**P value**	**miR**	**P value**	**miR**	**P value**
						mir-155	**0.003**
						mir-192	0.012
						mir-199b	0.05
						mir-204	0.003
						mir-221	1.03E-07
						mir-324-5p	1.55E-04
						mir-379	0.004
						mir-424	2.43E-04
						mir-429	2.82E-06
						mir-467b	1.29E-06
						mir-487b	1.87E-04
						mir-491	1.94E-04
						mir-497	4.75E-05
						mir-678	2.83E-07
						mir-680	1.23E-05
						mir-690	3.75E-06
						mir-692	2.16E-08
						mir-694	3.80E-09
						mir-706	3.60E-06
						mir-709	2.10E-04
						mir-721	0.009
